# Dietary Fiber Is Inversely Associated With Depressive Symptoms in Premenopausal Women

**DOI:** 10.3389/fnins.2020.00373

**Published:** 2020-05-06

**Authors:** Di Li, Yongqing Tong, Yan Li

**Affiliations:** Department of Clinical Laboratory, Renmin Hospital of Wuhan University, Wuhan, China

**Keywords:** fiber, depressive symptoms, premenopausal, perimenopausal, cross-sectional study

## Abstract

**Background:**

An inverse association between dietary fiber intake and depressive symptoms was reported in the general population, but this association is unstudied in midlife women. This study was designed to investigate the association of dietary fiber intake with depressive symptoms in midlife women.

**Methods:**

Analyses for this cross-sectional study were performed on baseline assessment of the Study of Women’s Health Across the Nation (SWAN). Linear regressions were used to examine the association of fiber intake with Center for Epidemiological Studies-Depression (CES-D) score. Logistic regression and restricted cubic spline analyses were used to examine the association between fiber intake and depressive symptoms (CES-D score ≥ 16).

**Results:**

A total of 3054 midlife women in our study were stratified into premenopausal women and early perimenopausal women by menstrual bleeding patterns. In premenopausal women, dietary fiber intake was inversely associated with CES-D scores in unadjusted, age-, education-, race/ethnicity-, total family income-, BMI-, sport-, use of antidepressant-, dietary total caloric intake-, SHBG-, and FSH-adjusted linear regression model. The fully adjusted regression coefficient with 95% confidence intervals (CIs) of fiber intakes was −0.146 (−0.235, −0.058) for CES-D score. Fiber intake was inversely associated with depressive symptoms (CES-D score ≥ 16) in crude and fully adjusted logistic regression model. The fully adjusted odds ratios (ORs) with 95% CI of depressive symptoms was 0.483 (0.314–0.745) in quartile 4 compared with quartile 1 for fiber intake. However, in early perimenopausal women, dietary fiber intake was not statistically significantly associated with depressive symptoms.

**Conclusion:**

Dietary fiber is inversely associated with depressive symptoms in premenopausal women, but not in early perimenopausal women.

## Introduction

Major depressive disorder is a chronic disease, characterized by low rates of remission and relatively high rates of relapse (Casacalenda et al., 2002). This chronic condition is expected to be a worldwide burden of disease, with approximately 300 million people affected (WHO, 2017; Li et al., 2018). Depression is expected to become the world’s leading cause of disability by 2030 (Mathers and Loncar, 2006; Ferrari et al., 2013). It is critical to investigate the modifiable risk factors and effective preventive methods for major depressive disorder.

Accumulating interest has focused on the relationships between dietary factors and depression in the last few years. According to the International Society for Nutritional Psychiatry Research, although the growing evidences linked to diet in depressive symptoms may be recent, it is now at a stage where it should be taken into account (Sarris et al., 2015). Epidemiological evidence showed that dietary factors, such as fish (Li et al., 2016), coffee (Wang et al., 2016), and vegetables (Liu et al., 2016), are related to the development of major depressive disorder. It has been found that the brain–gut axis, a bidirectional communication between the brain and the gut microbiota, plays a role in regulating brain function, via neural, immune, and endocrine pathway (Cryan and Dinan, 2012). The probiotics and gut microbiota could impact the levels of cytokines, which could affect function of brain (Cryan and Dinan, 2012). Moreover, the vagus and the tryptophan are related to relaying the effect of the intestinal microbiota to the brain (Cryan and Dinan, 2012). Conversely, the cortisol, regulated by hypothalamus–pituitary–adrenal axis, can influence immune cells, affect gut barrier function and permeability, and alter profile of intestinal microbiota (Cryan and Dinan, 2012). Dietary factors, especially fiber intakes from vegetables, fruits, and other plants, can change the composition of gut microbiota (Albenberg and Wu, 2014). However, few epidemiologic researches investigated the relationship between depressive symptoms and dietary fiber intake. A statistically significant inverse association between dietary fiber intake and depressive symptoms was found in an older (aged over 65) Chinese population (Woo et al., 2006) and in Japanese employees (Miki et al., 2016).

Nevertheless, associations of fiber intakes with depressive symptoms in midlife women are unstudied. Women in the midlife period would undergo dynamic changes in social roles and circumstances, such as marital disruption, the caring for aging family member, financial issues, death of a loved one, and children leaving or returning to the home, which may result in depressive symptoms (Rasgon et al., 2005). The changes in healthy status and health behaviors that happen during midlife also induce the development of depressive symptoms in women (Alexander et al., 2007; Gallicchio et al., 2007). Additionally, depression has long been associated with the menstrual cycle in women (Sassarini, 2016). In particular, women in perimenopausal period, when menstrual cycles become irregular, are at a higher risk of depressive symptoms than pre- or post-menopausal women (Parry, 2008). Therefore, it is possible that modifiable risk factors and preventive methods for depression in midlife women differ from those in other groups (Li et al., 2020). However, evidence for the associations of depressive symptoms with dietary fiber intake in midlife women are lacking.

Therefore, in the present study, analyses were conducted on the Study of Women’s Health Across the Nation (SWAN) to investigate the associations between daily dietary fiber intakes and depressive symptoms in midlife women.

## Materials and Methods

### Study Population and Data Collection

Analyses were conducted on the baseline data of the SWAN, a population-based and multicenter cohort of midlife women (Sowers et al., 2000). The cohort was recruited between 1995 and 1997 via a telephone screening interview to determine individual eligibility. Community-based samples of participants were obtained at seven sites across the United States using a variety of recruitment strategies and sampling frames. A total of 3302 women were included in the SWAN study. Women who met the eligibility criteria were 42–52 years old, had not used reproductive hormones in the last 3 months, had one or more menstrual period, and had an intact uterus and one or more intact ovary. The protocol of the SWAN was approved by the institutional review boards at all sites. Informed consent was obtained by all participants. The protocol contained extensive self-reported psychological symptoms, lifestyle, psychosocial, reproductive, physical, and health. Thirty-eight women without information of CES-D score, 164 women without information of diet, 51 women without information of menopausal status, and 5 women with hormone use were excluded. The final cohort in this research included 3054 women who provided fiber intake data and depression symptom data.

### Depression Assessment

Depressive symptoms were assessed with the 20-item Center for Epidemiological Studies Depression Scale (CES-D), a well-established measure with reliability in ethnically diverse populations that is widely used in epidemiological studies. The standard cutoff point for CES-D is 16, and a score of 16 or higher has been defined as indicative of clinically significant depressive symptomatology (Roberts, 1980).

### Dietary Intake Assessment

Dietary data were assessed at baseline via a modified 1995 Block interviewer-assisted food frequency questionnaire (FFQ) (Block et al., 1986; Subar et al., 2001) with 103 food items, on the basis of responses of Caucasians and African Americans in the Second National Health and Nutrition Examination Survey (NHANES) (Block et al., 1986, 1992; Centers for Disease Control and Prevention, 2002). DietSys software^1^ was used to assign nutrient values. These values were obtained from the United States Department of Agriculture (USDA) (1995) nutrient database for standard reference.

### Covariates

Demographic characteristics included age, race/ethnicity, education, and total family income. Healthy covariates included sport, menopausal status, body mass index (BMI), use of antidepressant, sex hormone binding globulin (SHBG), and follicle-stimulating hormone (FSH). Age, education, and race/ethnicity identification was based on self-reported identification. BMI was calculated as weight (kg) divided by height (meters) squared. Use of antidepressant, frequency of sport in past year, and current smoker were self-reported. SHBG and FSH in serum were detected using an ACS-180 analyzer (Bayer Diagnostics Corporation, Norwood, MA, United States). Menopausal status was classified into early perimenopausal (menstrual bleeding in the past 3 months accompanied by alters in cycle regularity in the past year) and premenopausal (menstrual bleeding in the past 3 months with no alter in cycle regularity in the past year) groups based on self-reported menstrual bleeding patterns (Harlow et al., 2007, 2008).

### Statistical Analysis

Analyses were conducted using R 3.5.2 and SPSS 20.0. Kolmogorov–Smirnov test showed that all continuous variables represent a non-normal distribution. Therefore, continuous variables were described by the median with the interquartile range (IQR). Mann–Whitney *U* and chi-square tests were conducted to compare the averages of continuous variables and percentages of categorical variables between women without depressive symptoms (CES-D score < 16) versus with depressive symptoms (CES-D score ≥ 16). Linear regression was used to examine the associations between CES-D scores and fiber intake. Fiber intake was categorized by quartiles. Logistic regression was performed to examine the relationships between fiber intakes and depressive symptoms, with quartile 1 as the reference category. The crude model had no adjustment. Model 1 was adjusted for use of antidepressant, dietary total caloric intake, BMI, sport, education, total family income, age, and race/ethnicity, and model 2 was adjusted for model 1 plus SHBG and FSH. To further investigate the associations of fiber intakes with depression symptoms, restricted cubic spline analysis was conducted in a fully adjusted model. A *p* value (two-tailed) of less than 0.05 was considered statistically significant.

## Results

### Sample Characteristics According to CES-D Score

Characteristics for 3054 participants by CES-D Score are presented in Table 1. There were significant differences between women with depressive symptoms and those without depressive symptoms in fiber intake, total calories intake, use of antidepressant, BMI, menopausal status, sport, total family income, education, race/ethnicity, and age. The dietary fiber intake in women without depressive symptoms was significantly higher than that in women with depressive symptoms (*p* < 0.001).

**TABLE 1:** Sample characteristics across by CES-D score.

Variable	Total (*n* = 3054)	CES-D score < 16 (*n* = 2308)	CES-D score ≥ 16 (*n* = 746)	*p* value
Age (years)	46(44−48)	46(44−48)	45(43−47)	<0.001
Race/ethnicity (%)				<0.001
Black/African American	848(27.8%)	615(26.6%)	233(31.2%)	
Chinese/Chinese American	241(7.9%)	207(9.0%)	34(4.6%)	
Japanese/Japanese American	245(8.0%)	207(9.0%)	38(5.1%)	
Caucasian/White Non-Hispanic	1469(48.1%)	1136(49.2%)	333(44.6%)	
Hispanic	251(8.2%)	143(6.2%)	108(14.5%)	
Education				<0.001
Less than high school	214(7.1%)	121(5.3%)	93(12.6%)	
High school graduate	531(17.5%)	360(15.7%)	171(23.1%)	
Some college/technical school	956(31.6%)	710(31.0%)	246(33.2%)	
College graduate	617(20.4%)	495(21.6%)	122(16.5%)	
Postgraduate	711(23.5%)	602(26.3%)	109(14.7%)	
Total family income				<0.001
Less than $19,999	432(14.5%)	233(10.3%)	199(27.9%)	
$20,000 to $49,999	1011(34.0%)	749(33.2%)	262(36.7%)	
$50,000 to $99,999	1089(36.7%)	883(39.1%)	206(28.9%)	
$100,000 or more	439(14.8%)	392(17.4%)	47(6.6%)	
Sport				<0.001
Never	809(26.8%)	528(23.1%)	281(38.2%)	
Less than once a month	394(13.0%)	291(12.7%)	103(14.0%)	
Once a month	163(5.4%)	127(5.6%)	36(4.9%)	
2–3 times a month	330(10.9%)	263(11.5%)	67(9.1%)	
Once a week	333(11.0%)	265(11.6%)	68(9.3%)	
More than once a week	994(32.9%)	814(35.6%)	180(24.5%)	
Menopausal Status				<0.001
Early perimenopausal	1403(45.9%)	1008(43.7%)	395(52.9%)	
Premenopausal	1651(54.1%)	1300(56.3%)	351(47.1%)	
FSH (mIU/ml)	15.80(10.70−26.35)	15.90(10.80−26.40)	15.50(10.41−26.10)	0.357
SHBG (nM)	40.90(28.00−57.50)	40.90(28.00−57.40)	40.85(27.80−58.10)	0.918
BMI (kg/m^2^)	26.68(22.92−32.14)	26.31(22.70−31.46)	28.15(23.67−33.98)	<0.001
Use of antidepressant				<0.001
No	2833(92.8%)	2197(95.2%)	636(85.3%)	
Yes	221(7.2%)	111(4.8%)	110(14.7%)	
Dietary total caloric intake (kcal/day)	1719.02(1351.93−2186.84)	1690.67(1333.40−2150.03)	1814.69(1414.96−2321.28)	<0.001
Dietary fiber intake (g/day)	11.37(8.32−15.06)	11.45(8.45−15.20)	11.17(7.99−14.69)	<0.001
CES-D score	8(3−15)	5(2−9)	23(18−30)	<0.001

*BMI, body mass index; CES-D, Center for Epidemiological Studies Depression; SHBG, sex hormone binding globulin; FSH, follicle-stimulating hormone.*

### Association Between Baseline Fiber Intake and CES-D Scores

The associations between dietary fiber intakes and CES-D scores are presented in Table 2. In premenopausal women, the regression coefficient with the 95% *CIs* of CES-D scores −0.084 (−0.161, −0.007) in the crude model indicated that fiber intake was inversely associated with the CES-D scores. After adjustment for use of antidepressant, dietary total caloric intake, BMI, sport, education, total family income, age, and race/ethnicity in model 1, high fiber intakes were significantly associated with lower CES-D scores, with a regression coefficient and 95% CI of −0.144 (−0.232, −0.056). This inverse association remained statistically significant when additionally adjusting for FSH and SHBG in model 2. The regression coefficient with the 95% CI was −0.146 (−0.235, −0.058) in model 2. However, in early perimenopausal women, dietary fiber intake was not statistically significantly associated with depressive symptoms.

**TABLE 2:** Associations between CES-D scores and dietary fiber intake (regression coefficient and 95% confidence intervals).

	Premenopausal			Early Perimenopausal		
		
	Standard Error	β (95% CI)	*p* value	Standard Error	β (95% CI)	*p* value
Crude	0.039	−0.084 (−0.161, −0.007)	0.034	0.048	−0.048 (−0.141, 0.045)	0.314
Model 1	0.046	−0.144 (−0.232, −0.056)	0.001	0.052	−0.043 (−0.145, 0.059)	0.409
Model 2	0.045	−0.146 (−0.235, −0.058)	0.001	0.052	−0.040 (−0.143, 0.062)	0.443

*Crude no adjustment. Model 1 adjusted for age, race/ethnicity, total family income, education, sport, BMI, dietary total caloric intake, and use of antidepressant. Model 2 adjusted for model 1 plus SHBG and FSH.*

### Association Between Baseline Fiber Intake and Depressive Symptoms

Table 3 presents the odds ratios (ORs) and 95% *CIs* of depressive symptoms (CES-D score ≥ 16) for the daily dietary fiber intake. In premenopausal women, the ORs of depressive symptoms in the crude model showed that fiber intake was inversely associated with depressive symptoms. After adjustment for use of antidepressant, dietary total caloric intake, BMI, sport, education, total family income, age, and race/ethnicity in model 1, the results were similar to those of the crude model. This association changed little and remained statistically significant when additionally adjusting for SHBG and FSH in model 2. The OR of depressive symptoms in model 2 was 0.483 (0.314–0.745) in quartile 4 (the highest) versus quartile 1 (the lowest) of the fiber intakes. However, in early perimenopausal women, dietary fiber intake was not statistically significantly associated with depressive symptoms.

**TABLE 3:** Odds ratios (95% confidence intervals) of depressive symptoms (CES-D score ≥ 16) across quartiles of dietary fiber intakes.

	Premenopausal				Early Perimenopausal			
		
	Cutoff	Crude	Model 1	Model 2	Cutoff	Crude	Model 1	Model 2
Fiber intake (g/day)								
Quartile 1 (low)	<8.39	1.000 (ref.)	1.000 (ref.)	1.000 (ref.)	<8.21	1.000 (ref.)	1.000 (ref.)	1.000 (ref.)
Quartile 2	8.39–11.43	0.775 (0.559–1.074)	0.670 (0.468–0.957)	0.670 (0.468–0.958)	8.21–11.31	0.794 (0.570–1.105)	0.804 (0.554–1.166)	0.801 (0.550–1.165)
Quartile 3	11.43–15.22	0.763 (0.550–1.059)	0.643 (0.442–0.936)	0.643 (0.442–0.937)	11.31–14.93	0.983 (0.711–1.357)	0.992 (0.679–1.449)	0.983 (0.671–1.441)
Quartile 4 (high)	≥15.22	0.705 (0.506–0.982)	0.497 (0.323–0.764)	0.483 (0.314–0.745)	≥14.93	0.842 (0.606–1.169)	830 (0.541–1.276)	0.849 (0.550–1.311)
*P*-trend		0.044	0.002	0.001		0.562	0.640	0.701

*Test for trend based on variable containing median value for each quintile. Crude no adjustment. Model 1 adjusted for age, race/ethnicity, total family income, education, sport, BMI, dietary total caloric intake, and use of antidepressant. Model 2 adjusted for model 1 plus SHBG and FSH.*

### Restricted Cubic Spline Analyses

**FIGURE 1:**
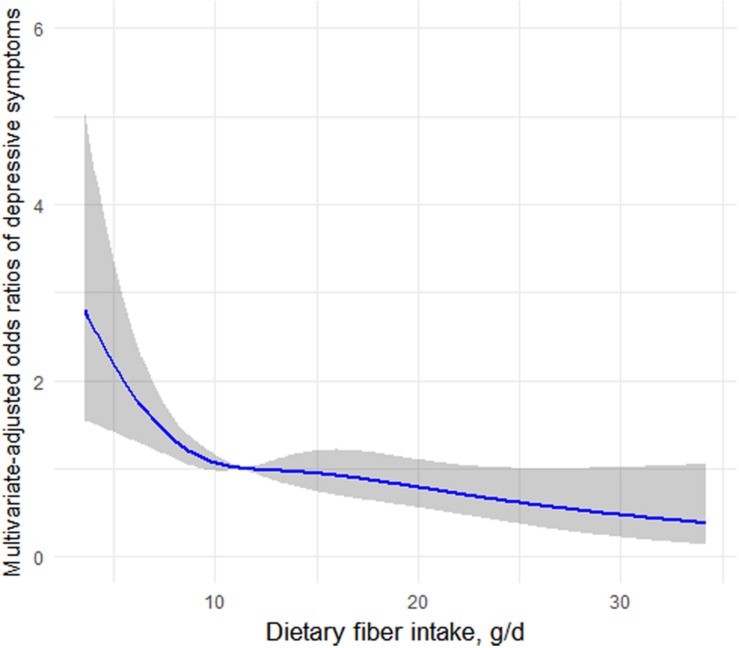
Restricted cubic spline analysis of the odds ratios of depressive symptoms with dietary fiber intakes in a fully adjusted model. The gray area represents the 95% CIs.

Figure 1 depicts the association of fiber intake with depressive symptoms determined using restricted cubic spline analyses in premenopausal women. The results, suggesting an L-shaped association, indicated that dietary fiber intake was dose–response inversely associated with prevalence of depressive symptoms (CES-D score ≥ 16) in premenopausal women.

## Discussion

In this study, analyses were conducted on the baseline data of SWAN and included 3054 midlife women aged 42–52 years to investigate the associations between depressive symptoms and dietary fiber intakes. We found that, in premenopausal women, dietary fiber intake was inversely associated with CES-D score, and these inverse associations of fiber intakes remained statistically significant after adjusting for potential confounders. Similar inverse associations were found between depressive symptoms and fiber intake in crude model, use of antidepressant-, dietary total caloric intake-, BMI-, sport-, education-, total family income-, age- and race/ethnicity-, SHBG-, and FSH-adjusted model in premenopausal women. However, dietary fiber intake was not statistically significantly associated with depressive symptoms in early perimenopausal women. To the best of our knowledge, this is the first time to explore the associations between fiber intake and depressive symptoms in midlife women.

Food is a primordial need for human well-being and survival. Nevertheless, food not only is vital to maintain health, human growth, and reproduction, but also supports and modulates our gut microbiota. Dietary fiber neither absorbed nor hydrolyzed in the intestine due to its resistance to the endogenous digestive enzymes (Jones, 2014). Dietary fiber can change the gut environment by supplying nutrition for gut microbial growth (Deehan et al., 2017). Intake of dietary fibers at low levels may partly result in the decrease of some bacterial taxa (Sonnenburg and Sonnenburg, 2014). These changes may lead to malfunctions, resulting in the development of chronic inflammatory diseases such as obesity, autoimmune diseases, allergies, colorectal cancer, and intestinal bowel disease (Sonnenburg and Sonnenburg, 2014). These diseases can be prevented by dietary fiber (Deehan and Walter, 2016). Recent studies indicated that the brain–gut axis, a bidirectional communication between the brain and the gut microbiota, acts as a pathway to adjust function of brain, via neural, immune, and endocrine pathways (Cryan and Dinan, 2012). Probiotics and the gut microbiota can impact the levels of cytokines, which may affect the function of brain (Cryan and Dinan, 2012). Dietary fiber intakes from fruits, vegetables, and other food, may change the composition of gut microbe (Albenberg and Wu, 2014). Therefore, dietary fiber may affect the development of depression via adjusting the gut microbiota.

However, few epidemiologic researches investigated the relationship between depressive symptoms and dietary fiber intake. A study that enrolled 3394 Chinese older adults found an inverse association between prevalence of depressive symptoms and dietary fiber intake (Woo et al., 2006). In a Japanese study among 1977 employees, dietary fiber intake from fruits and vegetables was significantly inversely associated with depressive symptoms (Miki et al., 2016). Nevertheless, associations of fiber intakes with depressive symptoms in midlife women are unstudied. Women in midlife would enter the menopausal transition period, which marks a peak time for decline of estrogen. With the alterations of endocrine, a variety of physiological changes, including gut microbiota, could arise in women in the menopausal transition period. While the expression of estrogen receptor-β (ERβ) is found in mouse and human colon epithelium (Enmark et al., 1997; Campbell-Thompson et al., 2001), a research in ERβ knock-out female mice indicated that ERβ alters gut microbe in a diet-specific manner (Menon et al., 2013). Additionally, since estrogens have been found to decrease food intake (Bless et al., 2014), it is not impossible that estrogens impact gut microbe by changing substrate availability. Estrogens can also function as substrates for gut microbe. For instance, gut microbes with β-glucosidase and β-glucuronidase enzymes, such as *Clostridium*, *Bifidobacterium*, and *Lactobacillus* spp., convert estrogens from inactive forms into their active forms by deconjugation (Flores et al., 2012; McIntosh et al., 2012; Kwa et al., 2016). Therefore, the decreased estrogen in midlife women who went through menopausal transition period could change gut microbiota. It is also possible that the associations between fiber intake and depressive symptoms could change in menopausal transition period women. Our study provided epidemiological evidence and found that fiber intake was not associated with depressive symptoms in early perimenopausal women. However, further studies will be needed to explore the underlying mechanism.

 However, when interpreting the founding of this research, some limitations should be taken into account. First, it is hard to make causal inferences due to a cross-sectional design. Therefore, it is possible that depression may lead to reduced food intake causing the decreased fiber intakes. Second, a paucity of quantitative assessment of fiber intake is another limitation. Assessment of dietary fiber intake information depended on the participant’s ability to remember, which might induce recall bias. Additionally, memory may be impacted by depressive symptoms, which may potentially result in assessment error. Finally, our results were controlled for a variety of important potential confounders; nevertheless, the presence of some unknown confounders and unmeasured confounders was hard to be excluded.

In summary, our results show that dietary fiber intake is inversely associated with depressive symptoms in premenopausal women, but not in early perimenopausal women. Prospective studies are needed to verify our findings.

## Data Availability Statement

The data of SWAN study are available from https://www.swanstudy.org/.

## Author Contributions

DL: data curation, methodology, writing – original draft, and writing – review and editing. YT: supervision and methodology. YL: funding acquisition, supervision, and validation.

## Conflict of Interest

The authors declare that the research was conducted in the absence of any commercial or financial relationships that could be construed as a potential conflict of interest.

## Footnotes

1 http://appliedresearch.cancer.gov/DietSys/software.html
